# NIA Genetics of Alzheimer’s Disease Data Storage Site (NIAGADS) AI‐Enhanced Search

**DOI:** 10.1002/alz.092685

**Published:** 2025-01-03

**Authors:** Emily Greenfest‐Allen, Otto Valladares, Prabhakaran Gangadharan, Heather White, Amanda B Kuzma, Pavel P. Kuksa, Wan‐Ping Lee, Yuk Yee Leung, Li‐San Wang

**Affiliations:** ^1^ Penn Neurodegeneration Genomics Center, Dept of Pathology and Laboratory Medicine, University of Pennsylvania, Philadelphia, PA USA; ^2^ Institute for Biomedical Informatics, Perelman School of Medicine, University of Pennsylvania, Philadelphia, PA USA; ^3^ University of Pennsylvania Perelman School of Medicine, Philadelphia, PA USA; ^4^ Penn Neurodegeneration Genomics Center, Perelman School of Medicine, University of Pennsylvania, Philadelphia, PA USA; ^5^ Institute on Aging, Perelman School of Medicine, University of Pennsylvania, Philadelphia, PA USA

## Abstract

**Background:**

NIAGADS is a national data repository that offers qualified investigators access to genomic data for Alzheimer’s disease (AD) and related dementia. In addition, NIAGADS has made substantial effort to curate, harmonize, standardize, and disseminate AD‐relevant variant, gene, and sequence annotations from publications, functional genomics datasets, and summary statistics deposited at NIAGADS. These results are made available to the public in a collection of interactive knowledgebases (AD Variant Portal, FILER Functional Genomics Repository, VariXam, Alzheimer’s GenomicsDB & Genome Browser), all of which are accessible programmatically via the NIAGADS API. However, as these offerings grow, navigating them can be challenging. Here, we introduce AI‐based enhancements to NIAGADS sites to help guide researchers and facilitate data discovery.

**Method:**

We leverage OpenAI’s generative AI to build and train three large language models (LLMs) based on NIAGADS documentation, step‐by‐step recipes for data‐access requests, subject‐specific vocabularies, and the OpenAPI specification defining the NIAGADS API that allows programmatic access to the NIAGADS knowledgebases. For users of the API and to enhance search interfaces, we build on the LLMs to construct a framework for handling complex natural language instructions that decomposes an inquiry into tasks and subtasks and then plans, selects, and optionally executes API calls and parses the results.

**Result:**

Developing these LLMs allows NIAGADS to improve user experiences by integrating topic‐specific chatbots and generative AI search tools into NIAGADS sites. Rule‐based chatbots that leverage conversational AI on the NIAGADS portal and Data Sharing Service will respond to inquiries with answers inferred from the LLMs, with responses improving with user feedback. These bots will also supplement help requests, suggesting solutions to common inquiries. Planner‐enhanced generative AI based on the API‐specification trained LLMs will be tied to knowledgebase searches and filters in resources such as the GenomicsDB and FILER to allow users to leverage natural language processing to ask sophisticated questions that require multiple API calls to resolve the answer.

**Conclusion:**

Introducing AI‐enhanced search creates an interactive opportunity for NIAGADS users to learn new information or discover resources and tools they can use to supplement their research, which, in turn, improves NIAGADS ability to support AD genetics research.

